# Two types of insoles design to influence running biomechanics in opposite directions and individual responses

**DOI:** 10.3389/fbioe.2025.1501627

**Published:** 2025-04-17

**Authors:** Aurélien Patoz, Loris Trastour, Cyrille Gindre, Bastiaan Breine, Thibault Lussiana

**Affiliations:** ^1^ Research and Development Department, Volodalen Swiss Sport Lab, Aigle, Switzerland; ^2^ Institute of Sport Sciences, University of Lausanne, Lausanne, Switzerland; ^3^ Laboratoire Interuniversitaire de Biologie de La Motricité, University Savoie Mont Blanc, Chambéry, France; ^4^ Research and Development Department, Volodalen, Chavéria, France; ^5^ MPFRPV, Université de Franche-Comté, Besançon, France; ^6^ Exercise Performance Health Innovation (EPHI) platform, Besançon, France; ^7^ Department of Movement and Sports Sciences, Ghent University, Ghent, Belgium

**Keywords:** extension insole, flexion insole, insole interventions, global running pattern, duty factor

## Abstract

**Introduction:**

Global running patterns vary along a spectrum defined by the degree of body verticality. This continuum ranges from extension (upright extended postures) to flexion (forward-leaning positions characterized by flexion at the hips and knees). Understanding these patterns is crucial for effective injury rehabilitation. Recent research has identified inefficiencies in vertical load management, leading to the development of extension- or flexion-based exercises. Insoles, while not typically designed for comprehensive extension or flexion adjustments, can complement these exercises. This study tested two novel insoles—extension and flexion—designed by a podiatrist based on principles such as higher shore values for enhanced extension increased thickness for greater flexion.

**Methods:**

Eighteen recreational runners ran at 12 km/h on a treadmill under three conditions: no insole, extension insole, and flexion insole. We hypothesized that the extension insole would produce a lower duty factor (DF), greater vertical center of mass displacement (∆COM), and shorter time to maximum ankle pronation during ground contact ( $t_{\max.\;\text{pron}}$ ) with opposite effects expected for the flexion insole.

**Results:**

However, the results did not support this hypothesis, as no significant effects of either insole were observed on DF, ∆COM, or $t_{\max.\;\text{pron}}$ compared to running without an insole (*p* ≥ 0.38). Additionally, there was considerable variation in individual responses to the insoles. The extension insole resulted in a more extended running pattern in 50% of participants, while the flexion insole produced a more flexed pattern in 44% of participants. Notably, only 11% of participants reported both a more extended running pattern with the extension insole and a more flexed running pattern with the flexion insole.

**Discussion:**

The anticipated effects of the insoles on running mechanics were not consistently observed, underscoring the complexity of insole interventions. This highlights the need for further research to improve insole design, refine insole prescription, and to better understand the nuances of running biomechanics.

## Introduction

A diverse array of global running patterns spans a continuum of body verticality (Lussiana et al., 2019; Patoz et al., 2020; van Oeveren et al., 2021; Patoz et al., 2022b). Running gaits that are vertical or in extension show a forefoot strike pattern, high placed center of mass (COM) at contact and at mid stance, pronounced vertical oscillation, and very spring-like running gait with a low duty factor (DF) and high leg stiffness (Arendse et al., 2004). On the other side of the spectrum, running gaits that are more horizontal or in flexion show a rearfoot strike pattern, a lower placed COM at touchdown and at mid stance, and less vertical oscillation with a higher DF and lower leg stiffness (McMahon et al., 1987; Bonnaerens et al., 2019; Davis et al., 2020). Among these biomechanical variables, the DF and vertical COM displacement within a running step (∆COM) can be considered as the main global biomechanical variables that characterize the overall running pattern (Lussiana et al., 2019; Patoz et al., 2020). On the local level (i.e., the foot), the time to maximum ankle pronation during ground contact ( $t_{\max.\;\text{pron}}$ ) reflects a specific and important aspect of the running pattern. Midfoot strikers (with an extended ankle–in extension) exhibited shorter $t_{\max.\;\text{pron}}$ than rearfoot strikers (with a flexed ankle–in flexion) (Breine et al., 2017). No single pattern within this spectrum has proven superior in terms of running endurance performance or running-related injury risk (Moore, 2016; Lussiana et al., 2017; Hanley et al., 2019; Jauhiainen et al., 2020; Patoz et al., 2022a). Recognizing the significance of these global patterns, as opposed to solely focusing on injury location or specific biomechanical variables, is essential for effective injury prevention and rehabilitation strategies (Jauhiainen et al., 2020).

Running-related injuries can render any running pattern suboptimal, particularly in the management of vertical load—a critical factor given the weight-bearing nature of running (Breine et al., 2017; Ceyssens et al., 2019). Efficiency in running patterns relies on a delicate balance between compliance, which involves the acceptance of joint deformation, and stiffness, the resistance against such deformation (Gerritsen et al., 1995; Ahn et al., 2014; Stearne et al., 2014; Breine et al., 2017; Lussiana et al., 2017; Gindre et al., 2022). Building on this understanding, a recent approach has identified inefficiencies in vertical load management, distinguishing them as either ‘too soft’ (excessive compliance) or ‘too hard’ (excessive stiffness) (Gindre et al., 2022). Kinematic, kinetic, and spatiotemporal risk factors, as outlined in a systematic review by Ceyssens et al. (2019), were categorized according to these too soft or too hard inefficiencies. In response to these inefficiencies, a novel approach proposed prescribing extension- or flexion-based exercises tailored to address the specific vertical load management inefficiency (for detailed exercises, refer to Figure 2 in Gindre et al. (2022)). These exercises should actively influence the body to enhance its extension or flexion in the direction opposite to the identified inefficiency. For instance, prescribing extension-based exercises is suitable when there is excessive compliance (too soft), whereas flexion-based exercises are recommended in cases of excessive stiffness (too hard). A runner that overstrides can be defined as having a too hard running pattern (Ceyssens et al., 2019). In contrast, inconsistencies in mobilities between transverse and coronal plane motion (especially at the feet, knees, and hips) conceptually underpin the too soft running form (Buist et al., 2009; Ferber et al., 2010).

Alongside the proposed exercises, a complementary approach to load management could focus on the interaction between our feet and the ground, suggesting the use of specific shoe insoles. This combined approach aims to address vertical load inefficiencies more comprehensively. Insoles, extensively utilized in running (Kirby, 2017) have been shown to modify the vertical load but were not typically designed to address extension or flexion in a global context (Wilkinson et al., 2018; Van Alsenoy et al., 2019). Insoles exert their influence by modifying foot movements and altering mechanical constraints due (Frederick, 1986) to their biomechanical impact during running. Therefore, podiatric treatment involving insoles could effectively complement extension- or flexion-based exercises. The insoles should be designed to complement these exercises, fostering a running pattern that emphasizes either extension or flexion. Such insole design is grounded in evidence-based principles reported by podiatrists. Two key principles guide the creation of customized insoles. The first principle pertains to the general hardness of the insole, which influences biomechanical function. Higher shore values promote a more dynamic response, whereas lower shore values enhance anchoring, as demonstrated by impact tester measurements showing reduced and temporally delayed peak impact forces (Cook et al., 1985; Aerts and De Clercq, 1993; Shorten and Mientjes, 2011; Yang et al., 2024). This principle can be exemplified in athletic footwear: track spikes, with their rigid construction and minimal cushioning, are designed to support a dynamic, extension-focused running gait. Conversely, long-distance running shoes feature softer midsoles that enhance cushioning, aligning with the somewhat less dynamic requirements of endurance running compared to sprinting (Logan et al., 2010; Kettner et al., 2025). The second principle involves creating localized contrasts in hardness and thickness within the insole rather than altering its overall rigidity. Softer and lower zones are strategically placed to encourage foot movement toward these areas, while harder and elevated zones are designed to oppose the foot movement in this direction. A common example is the use of rigid postings to limit overpronation (Johanson et al., 1994; Fong et al., 2008; Costa et al., 2021). An important consideration when applying these principles is the thickness, or “stack height”, of the materials used. Soft materials, intended to enhance cushioning and anchoring, require sufficient thickness to accommodate deformation (Sun et al., 2008). If a soft material is too thin (e.g., 1 mm), it may compress fully and lose its functional capacity–a phenomenon known as “bottoming out” (Shorten, 1993). This effect is particularly pronounced in highly compliant materials. At the global biomechanical level, an extension-matched insole should result in a more extended lower limb, decreasing DF and increasing vertical COM displacement (∆COM) (Lussiana et al., 2019; Patoz et al., 2020). Conversely, a flexion-matched insole should induce a more flexed lower limb, increasing DF and reducing ∆COM (Lussiana et al., 2019; Patoz et al., 2020). On the local level, an extension insole should shorten $t_{\max.\;\text{pron}}$ whereas the flexion insole should lead to a longer $t_{\max.\;\text{pron}}$ .

This study aimed to test two distinct novel insoles (extension and flexion) designed to favor either extension or flexion during running. Insoles were designed by a podiatrist given creative freedom in their development but guided by the two key principles described above. We hypothesized that the extension insole would induce a lower DF, larger |∆COM|, and shorter $t_{\max.\;\text{pron}}$ compared to running without an insole. Conversely, we hypothesized that the flexion insole would induce a higher DF, smaller |∆COM|, and longer $t_{\max.\;\text{pron}}$ compared to running without an insole. Additionally, this study explored individual responses to the extension and flexion insoles. Understanding these individual variations is crucial, as responses to insoles can differ widely among runners due to unique biomechanical and physiological factors.

## Materials and methods

### Participants

Eighteen recreational endurance runners with regular running training, 14 males (variable: mean ± standard deviation, age: 32 ± 10 years, body mass: 72 ± 10 kg, height: 180 ± 6 cm) and four females (age: 31 ± 7 years, body mass: 60 ± 5 kg, height: 168 ± 2 cm) participated in the present experiment. To ensure diverse participation in the study, we sought a heterogeneous panel of runners with varying training backgrounds and running techniques (i.e., without a defined foot-strike pattern, cadence, or running style), aiming to capture most of the extension-flexion continuum. Consequently, participants were required to run a minimum of 1 hour per week, be in good self-reported general health with no current or recent (<3 months) musculoskeletal injuries, not wear any orthotics, and have a shoe size between 37 and 45 to ensure compatibility with the insoles (insoles are further described in Subsec. Insole design). The study protocol was approved by the Ethics Committee of the Vaud canton (commission cantonale d’éthique de la recherche sur l’être humain CER-VD 2020–00334) prior to participant recruitment. The protocol was conducted in accordance with international ethical standards (Harriss et al., 2017) and adhered to the latest Declaration of Helsinki of the World Medical Association.

### Insole design

**FIGURE 1:**
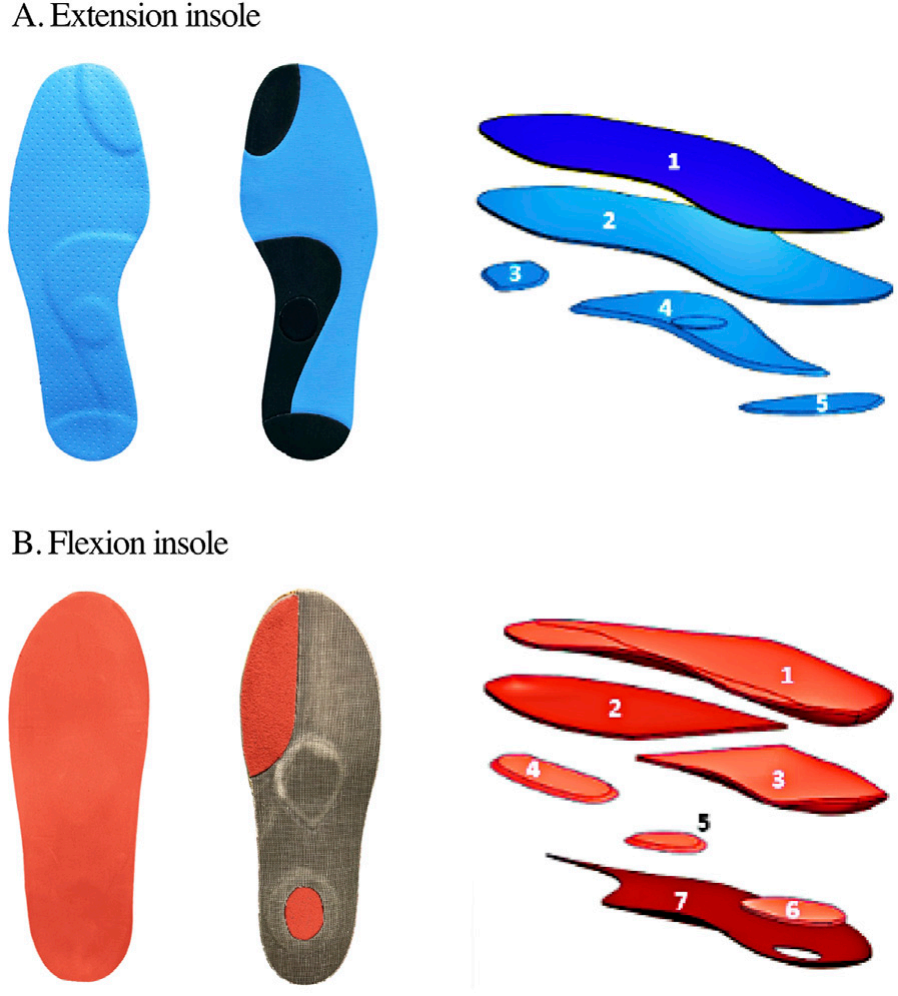
Top and bottom views as well as the different layers (1 up to 7) used to create **(A)** the extension insole and **(B)** the flexion insole. The material specifications of each layer are given in Table 1.

A podiatrist was instructed to create an extension insole (Figure 1A; Table 1), i.e., an insole that would promote more extension of the lower limb during running. The same podiatrist was also instructed to create a flexion insole (Figure 1B; Table 1), i.e., an insole that would promote more flexion of the lower limb during running. The podiatrist had creative freedom in crafting both extension and flexion insoles, guided by the two key principles described above. Insoles were produced in a range of sizes from 37 to 45. As there is currently no scientific consensus on the optimal materials for insole construction, their selection often depends on the individual practitioner’s experience. In this study, the material choices were guided by the established practices taught during the podiatrist’s university training. While recognizing the value of a more standardized approach, this methodology reflects common clinical practices and experience within the podiatric field, where decisions are frequently made based on the practitioner’s expertise. A single practitioner was involved in the design process to maintain consistency across conditions, particularly as the effects of insoles on running biomechanics may vary depending on the practitioner (Chevalier and Chockalingam, 2012).

**TABLE 1:** Material specifications of each layer (1 up to 7) of the extension and flexion insoles.

Insole	Properties	Layer 1	Layer 2	Layer 3	Layer 4	Layer 5	Layer 6	Layer 7
Extension	Material	PE/EVA	PE	EVA	EVA	EVA		
	Density (kg/m^3^)	250	360	360	360	360		
	Hardness (shore A)	35	70	70	70	70		
	Thickness (mm)	0.8	1	1	2	1		
Flexion	Material	PE/EVA	PE/EVA	EVA	PU	PU	PU	Flux Pro
	Density (kg/m^3^)	120	250	130	220	300	220	900
	Hardness (shore A)	25	40	30	8	10	8	90
	Thickness (mm)	2	2	2	2	2	2	0.8

Note. EVA: ethylene-vinyl acetate, PE: polyethylene, PU: polyurethane.

### Experimental procedure

Each participant completed one experimental session in the laboratory. After providing written informed consent, retro-reflective markers were positioned on participants (described in Subsec. Data collection and processing) to assess their running biomechanics. As for each participant, a 5-min warm-up run was performed on a motorized treadmill at 12 km/h (Medic 2,850, Technologies Machines Spéciales, Champs-sur-Yonne, France). This was followed, after a short break (<5 min) during which the stock insoles (factory insoles) were removed from the shoes of the participant, by a 5-s standing static trial on the same treadmill for calibration. Then, three 1-min runs at 12 km/h [no insole (no stock insole), extension insole, and flexion insole] were performed in a randomized order (5-min recovery period between each run, during which the adjustment of shoe insoles was done blindly with respect to the participant). Only the acute effects of the insoles on running biomechanics were observed during these short-duration runs. A speed of 12 km/h was chosen because it represents the average preferred pace of male runners (Selinger et al., 2022). Three-dimensional (3D) kinematic data were collected during the last 30 s of the running trial (82 ± 5 running steps), resulting in at least 25 steps being analyzed (Oliveira and Pirscoveanu, 2021). All participants were familiar with running on a treadmill as part of their usual training program and wore their habitual running shoes during testing. While not standardizing footwear could introduce potential confounding effects, as differences in shoe characteristics can influence running biomechanics (Sinclair et al., 2016), it was preferred to ensure comfort for the recreational runners, as they are generally more at ease wearing their own shoes (Hébert-Losier et al., 2020).

### Data collection and processing

Whole-body 3D kinematic data were collected at the maximal sampling frequency of our operating system (179 Hz) using eight infrared Oqus 500+ cameras and the Qualisys Track Manager software version 2022.2, build 7,710 (Qualisys AB, Göteborg, Sweden). The laboratory coordinate system was oriented such that the *x*-, *y*-, and *z*-axis denoted the medial-lateral (pointing towards the right side of the body), posterior-anterior, and inferior-superior axis, respectively. Sixty-six and sixty-four retro-reflective markers of 12 mm in diameter were used for static and running trials, respectively. They were affixed to the skin and shoes of individuals over anatomical landmarks using double-sided tape, following standard guidelines from the Project Automation Framework Running package (Tranberg et al., 2011), and as already reported in Lussiana et al. (2019).

The 3D marker data were exported in.c3d format and processed in Visual3D Professional software version 2021.01.1 (C-Motion Inc., Germantown, Maryland, USA). More explicitly, the 3D marker data were low-pass filtered at 20 Hz using a fourth-order Butterworth filter. From the marker set, a full-body biomechanical model with 13 rigid segments was constructed, with each segment tracked using six degrees of freedom. Segments included the head, upper arms, lower arms, thorax, pelvis, thighs, shanks, and feet. In Visual3D, segments were treated as geometric objects. Segments were assigned inertial properties and center of mass (COM) locations based on their shape (Hanavan, 1964) and were attributed a relative mass based on standard regression equations (Dempster, 1955). Whole-body COM location was calculated from the parameters of all 13 segments. Kinematic variables were calculated using rigid-body analysis and whole-body COM location was calculated from the parameters of all 13 segments (COM was directly provided by Visual3D). The ankle joint angle was defined as the orientation of the shank segment relative to the foot segment (distal relative to proximal) (Woltring, 1991) and was computed using a *y–x–z* Cardan sequence.

### Data analysis

Foot-strike and toe-off running events were derived from the accelerations and trajectories of the 3D heel marker and mid-toe landmark data using similar procedures to those previously reported and validated against an instrumented treadmill (gold standard method) (Patoz et al., 2021). Mid-stance and mid-flight running events were defined as the instant when the COM reaches its lowest and highest vertical position between two consecutive foot-strikes, respectively.

Ground contact time (*t*_*c*_) and swing time (*t*_*s*_) were defined as the time from foot-strike to toe-off and from toe-off to foot-strike of the same foot, respectively. These timings permitted to calculate DF as $\text{DF}=\frac{t_{c}}{t_{c}+t_{s}}$ (Minetti, 1998).

∆COM was calculated as the difference between the COM at mid-stance and mid-flight, leading to negative values. Hence, the extension insole should give larger |∆COM| than no insole. ∆COM was normalized by the average value of the COM of the standing static trial.

$t_{\max.\;\text{pron}}$ was given as the time elapsed between the foot-strike and the time to maximum ankle pronation during *t*_*c*_. The *y*-component of the ankle joint angle was used to determine the maximum ankle pronation during *t*_*c*_. First, the average ankle joint angle of the standing static trial was subtracted from that of running trials. Then, maximum ankle pronation during *t*_*c*_ was given by the negative of the minimum of the well of the *y*-component of the rescaled ankle joint angle observed during *t*_*c*_. The minimum of this signal represents the minimum ankle supination and therefore corresponds to the maximum ankle pronation.

For the three biomechanical measures (DF, ∆COM, and $t_{\max.\;\text{pron}}$ ), the values extracted for each participant were averaged over the 30 s run and over the right and left steps for subsequent analyses. As for each biomechanical measure, deviations (∆) between extension insole and no insole as well as between flexion insole and no insole were computed. Furthermore, to investigate the overall effect of the insole (∆ overall), each ∆ was rescaled between −0.5 and 0.5 but keeping their initial sign (positive or negative). Then, averaging these three ∆s leads to ∆ overall. A negative ∆ (∆ of any of the three biomechanical measures or ∆ overall) corresponds to a running pattern with more extension with the given insole than without insole. On the contrary, a positive ∆ corresponds to a running pattern with more flexion with the given insole than without insole. Finally, the number of participants and corresponding percentages were reported for whom the extension insole resulted in a negative deviation across each of the three biomechanical measures (∆ DF, ∆ ∆COM, and ∆ $t_{\max.\;\text{pron}}$ ), as well as for the overall deviation (∆ overall), and similarly for the flexion insole but for positive deviations. No threshold value was used to classify a deviation as negative or positive. Additionally, ∆ overall was used to report the number of participants and corresponding percentage who exhibited both a more extended running pattern with the extension insole and a more flexed running pattern with the flexion insole. Data analysis was performed using Python (v3.8.16, retrieved from http://www.python.org).

### Statistical analysis

The sample size was determined based on prior experience and is comparable to those used in Van Alsenoy et al. (2021) and Wilkinson et al. (2018). All data are presented as the mean ± standard deviation. Data normality was verified using Kolmogorov–Smirnov test (*p* ≥ 0.25). The effect of the extension and flexion insoles compared to no insole on the biomechanical measures were investigated using paired Student’s *t*-tests. In addition, correlations among the ∆s of the biomechanical measures (∆ DF, ∆ ∆COM, and ∆ $t_{\max.\;\text{pron}}$ ) were computed for both extension and flexion insoles to assess whether these parameters are interrelated within each type of insole. Very high, high, moderate, low, and negligible correlations were given by |*r*| values of 0.90–1.00, 0.70–0.89, 0.50–0.69, 0.30–0.49, and 0.00–0.29, respectively (Hinkle et al., 2002). Statistical analysis was performed using Jamovi (v1.6.23, retrieved from https://www.jamovi.org) and Python with a level of significance set at *p* ≤ 0.05.

## Results

### Mean biomechanical comparison between extension or flexion insoles and no insole

There was no significant effect of the extension insole compared to no insole (*p* ≥ 0.39; Table 2) and the differences were −0.13% ± 0.63% for DF, −0.01% ± 0.28% for ∆COM, and −0.4 ± 2.6 m for $t_{\max.\;\text{pron}}$ . Similarly, there was no significant effect of the flexion insole compared to no insole (*p* ≥ 0.38; Table 2) and the differences were 0.02% ± 0.6% for DF, −0.05% ± 0.30% for ∆COM, and 0.8 ± 3.5 m for $t_{\max.\;\text{pron}}$ .

**TABLE 2:** Biomechanical measures obtained when using no insole, the extension insole, and the flexion insole. No significant difference (*p* ≤ 0.05) was identified by paired Student’s *t*-tests between the extension insole and no insole and between the flexion insole and no insole.

Insole	DF (%)	∆COM (%)	$t_{\max.\;\text{pron}}$ (ms)
No insole	36.2 ± 3.0	−8.35 ± 1.01	73.4 ± 9.0
Extension	36.1 ± 3.1	−8.36 ± 1.10	73.0 ± 9.2
Flexion	36.2 ± 3.1	−8.41 ± 1.16	74.1 ± 7.1
Extension vs. no insole (*p*)	0.39	0.88	0.54
Flexion vs. no insole (*p*)	0.92	0.46	0.38

Note. Data are given as mean ± standard deviation. Duty factor: DF, vertical center of mass displacement: ∆COM, and time to maximum ankle pronation during ground contact: *t*_max. pron_.

### Correlations among the deviations of the biomechanical measures

Correlation between ∆ DF and ∆ ∆COM was significantly positive and moderate and high for the extension and flexion insoles, respectively (*r* ≥ 0.60; *p* ≤ 0.008; Table 3). Correlations between ∆ COM and ∆ $t_{\max.\;\text{pron}}$ was non-significant and negligible for the extension insole (*r* = 0.15; *p* = 0.54; Table 3) and significantly positive and moderate for the flexion insole (*r* = 0.48; *p* = 0.04; Table 3). Correlation between ∆ COM and ∆ $t_{\max.\;\text{pron}}$ was non-significant and negligible and low for the extension and flexion insoles, respectively (*r* ≤ 0.44; *p* ≥ 0.07; Table 3).

**TABLE 3:** Pearson’s correlation coefficients (*r*) and corresponding *p*-values among the deviations (∆) of the three biomechanical measures for both extension and flexion insoles compared to no insole. Significant correlations (*p* ≤ 0.05) are depicted in bold.

Insole	Biomechanical measures	∆ ∆COM		∆ $t_{\max.\;\text{pron}}$	
		*r*	*p*	*r*	*P*
Extension	∆ DF	**0.60**	**0.008**	0.15	0.54
	∆ ∆COM			−0.06	0.82
Flexion	∆ DF	**0.81**	**<0.001**	**0.48**	**0.04**
	∆ ∆COM			0.44	0.07

Note. Duty factor: DF, vertical center of mass displacement: ∆COM, and time to maximum ankle pronation during ground contact: *t*_max. pron_.

### Individual responses to extension and flexion insoles compared to no insole

**FIGURE 2:**
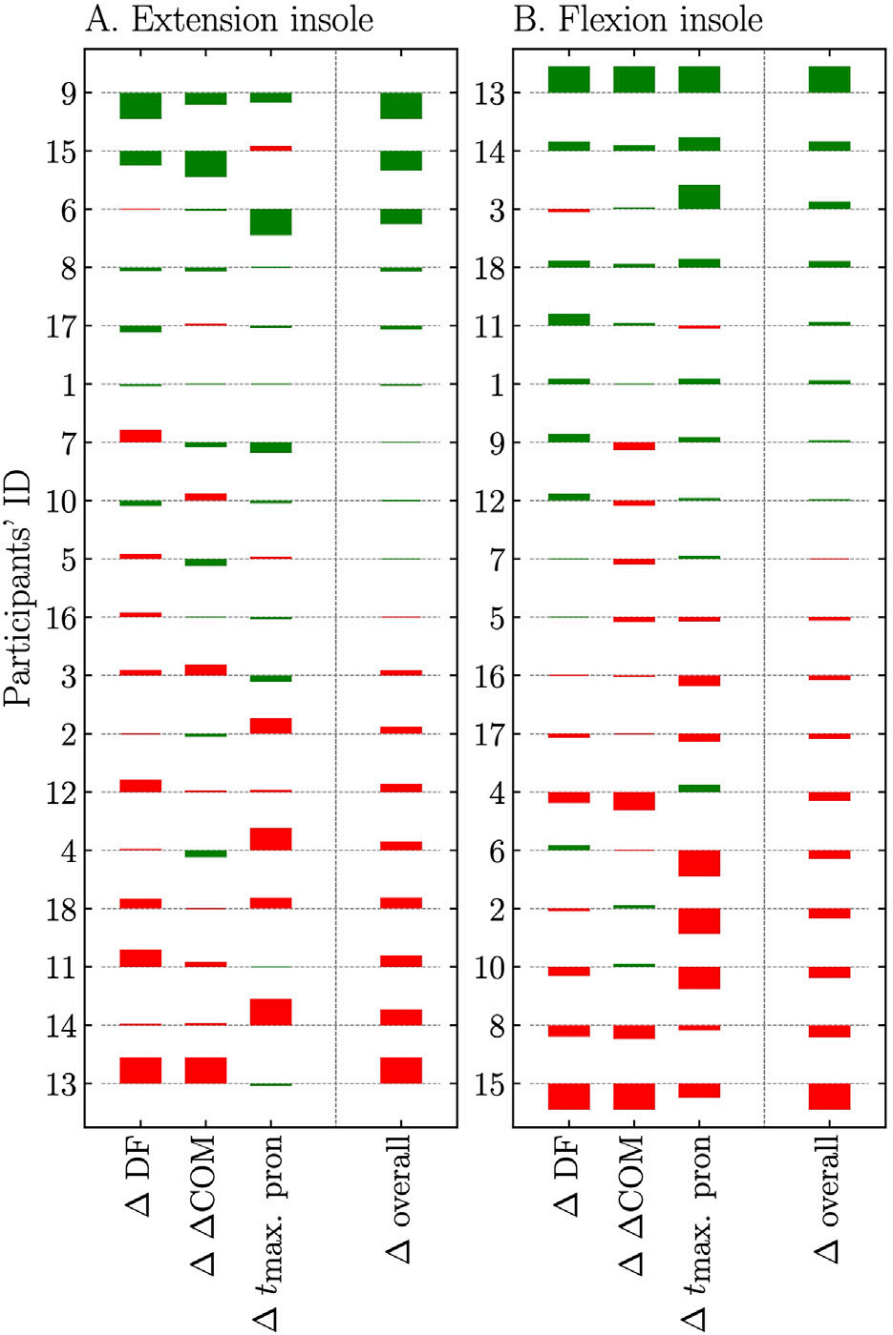
Deviations (∆) rescaled between −0.5 and 0.5 but keeping their initial sign (positive or negative) of the biomechanical measures (duty factor: DF, vertical center of mass displacement: ∆COM, and time to maximum ankle pronation during ground contact: *t*_max. pron_) for **(A)** the extension insole and **(B)** the flexion insole. ∆ overall, calculated as the mean ∆ of the three biomechanical measures, is also provided for both extension and flexion insoles. Data are ordered from largest (top) to lowest (bottom) expected effect according to ∆ overall value for both the extension and flexion insoles (participants’ ID). Green color is used when ∆ of the biomechanical measure is in accordance with the expected change provided by the insole (decrease and increase with extension and flexion insole, respectively) while red color is used otherwise.

As for the extension insole, the average deviations were −0.13% ± 0.62% for ∆ DF, −0.01 ± 0.28 cm for ∆ ∆COM, and −0.4 ± 2.6 m for ∆ $t_{\max.\;\text{pron}}$ (Table 4). The flexion insole reported average deviations of 0.02% ± 0.66%, −0.05 ± 0.30 cm, and 0.8 ± 3.5 m for ∆ DF, ∆ ∆COM, and ∆ $t_{\max.\;\text{pron}}$ , respectively (Table 4). The extension insole aligned with the podiatrist’s design intentions, i.e., resulted in a negative deviation for DF, ∆COM, and $t_{\max.\;\text{pron}}$ in 6 (33%), 10 (56%), and 11 (61%) participants, respectively (Table 4; Figure 2). Conversely, the flexion insole aligned with the podiatrist’s design intentions, i.e., produced a positive deviation for DF, ∆COM, and $t_{\max.\;\text{pron}}$ in 10 (56%), 8 (44%), and 9 (50%) participants, respectively (Table 4; Figure 2). ∆ overall, which represents the average of the three biomechanical measures, revealed that the extension insole induced a more extended running pattern in nine participants (50%), while the flexion insole resulted in a more flexed running pattern in eight participants (44%; Figure 2). Only two participants (11%; ID 9 and 1; Figure 2) reported both a more extended running pattern with the extension insole (negative ∆ overall) and a more flexed running pattern with the flexion insole (positive ∆ overall).

**TABLE 4:** Deviations (∆) of the biomechanical measures (DF, ∆COM, and *t*_max. pron_) for the extension and flexion insoles. Data are ordered from largest (top) to lowest (bottom) expected effect according to ∆ overall value (participants’ ID). Green color is used when ∆ of the biomechanical measure is in accordance with the expected change provided by the insole (decrease and increase with extension and flexion insole, respectively) while red color is used otherwise.

Extension insole				Flexion insole			
ID	∆ DF (%)	∆ ∆COM (cm)	∆ $t_{\max.\;\text{pron}}$ (ms)	ID	∆ DF (%)	∆ ∆COM (cm)	∆ $t_{\max.\;\text{pron}}$ (ms)
9	-2.07	-0.31	-3.0	13	1.44	0.75	8.4
15	-1.15	-0.67	0.7	14	0.52	0.17	4.4
6	0.01	-0.04	-7.8	3	-0.19	0.05	7.9
8	-0.30	-0.10	-0.0	18	0.36	0.10	2.8
17	-0.54	0.05	-0.7	11	0.66	0.07	-0.4
1	-0.16	-0.01	-0.3	1	0.30	0.01	1.8
7	0.30	-0.12	-3.1	9	0.46	-0.21	1.6
10	-0.44	0.20	-0.8	12	0.38	-0.14	0.9
5	0.11	-0.18	0.3	7	0.01	-0.15	1.0
16	0.11	-0.01	-0.6	5	0.01	-0.13	-0.6
3	0.13	0.30	-1.9	16	-0.02	-0.04	-1.5
2	0.03	-0.08	2.2	17	-0.23	-0.04	-1.2
12	0.29	0.04	0.3	4	-0.67	-0.50	2.3
4	0.03	-0.18	3.2	6	0.28	-0.02	-3.9
18	0.23	0.01	1.6	2	-0.17	0.10	-3.8
11	0.41	0.14	-0.1	10	-0.56	0.10	-3.3
14	0.04	0.06	3.7	8	-0.69	-0.38	-0.8
13	0.61	0.72	-0.7	15	-1.60	-0.71	-2.1

Note. Duty factor: DF, vertical center of mass displacement: ∆COM, and time to maximum ankle pronation during ground contact: *t*_max. pron_. ∆ overall is calculated as the mean ∆ of the three biomechanical measures rescaled between -0.5 and 0.5 but keeping their initial sign (positive or negative).

## Discussion

Contrary to our initial hypotheses, the present study did not find conclusive evidence supporting the anticipated effects of the extension and flexion insoles on running biomechanics. The highly variable individual responses to the extension and flexion insoles underline the intricate nature of insole interventions. These findings therefore question the efficacy of these specific insoles in inducing the desired alterations in running patterns.

The extension/flexion insoles did not induce a lower/higher DF, larger/smaller |∆COM|, and shorter/longer $t_{\max.\;\text{pron}}$ compared to no insole (*p* ≥ 0.38; Table 2). This lack of significant impact aligns with the broader literature, suggesting that insoles may not consistently yield substantial effects. For instance, the effect of medial foot orthoses on eversion and tibial rotations were found to be small and non-systematic over subjects (Stacoff et al., 2000). Similarly, individual movement changes of the path of the center of pressure were small and non-systematic between a neutral insert and four different inserts, and reactions were not consistent between subjects (Nigg et al., 2003). Besides, compared with the neutral insert condition, subjects showed increases or decreases of their knee joint moments (Nigg et al., 2003). A recent study on adults with excessive foot pronation reported that 6° and 9° medial wedge insoles decreased the ankle eversion angle during early stance and increased this angle during the propulsive phase, but the 3° medial wedge insole did not yield any effect (Costa et al., 2021). Altogether, the observed very weak effect questions the efficacy of specific insoles in inducing the desired alterations in running patterns.

The present study did report significant moderate and high correlations between the deviations of the two global variables (∆ DF and ∆ ∆COM; *r* ≥ 0.60; *p* ≤ 0.008; Table 3) for both insoles. However, this study did not establish a clear correlation between the deviations of the two global variables and the local variable (∆ $t_{\max.\;\text{pron}}$ ; *r* ≤ 0.44; *p* ≥ 0.07; Table 3), except between ∆ COM and ∆ $t_{\max.\;\text{pron}}$ for the flexion insole (moderate correlation; *r* = 0.48; *p* = 0.04; Table 3). Hence, the deviation in the local variable (∆ $t_{\max.\;\text{pron}}$ ) reflecting changes in local (foot-level) flexion or extension, was not related to the deviations in the global variables (∆ DF, ∆ ∆COM), which represent changes in whole-body (COM) flexion or extension. These findings suggest a dissociation between changes in the global running pattern and specific localized effects. This underscores the complexity of the biomechanical interactions in running and raises questions about how local and global adaptations to insole interventions interact. We recommend that future studies examine both local and global effects in greater detail to better understand this complexity. Such insights could enable more individualized insole designs, optimizing their effectiveness for different runners. Despite significant advancements in footwear technology—such as various cushioning systems in modern running shoes—the interactions between these technologies and local versus global biomechanics remain poorly understood.

The complexity of insole prescription is evident in our study and the two key principles used to design the insoles aiming at modifying running patterns might be questioned. However, prior research indicated that an augmentation in midsole thickness resulted in both a greater knee-flexion at foot-strike and a larger foot-strike angle (more rearfoot) (Law et al., 2019; Mai et al., 2023). Similarly, medial wedges and dual hardness insoles were demonstrated to reduce foot pronation angle during the running stance phase (Oh et al., 2017; Braga et al., 2019). This suggests that the variety of components employed in the extension and flexion insoles (Figure 1; Table 1) might have played a role, as an amalgamation of properties, rather than a single specific property, could impact the material behavior. Moreover, variations in the interpretation and application of these two principles by different podiatrists might lead to differences in effects observed. This might highlight the need to question and thoroughly understand the specific biomechanical mechanisms underlying the concepts incorporated into insoles, as well as exploring potential differences in the implementation of these concepts by different practitioners.

The average deviations were small with large standard deviations for the three biomechanical measures (e.g., ∆ DF: 0.13% ± 0.62% and 0.02% ± 0.66% for the extension and flexion insole, respectively), highlighting the considerable variability in individual responses. This justifies the exploration of the individual responses to the extension and flexion insoles in our analysis. Some individuals depict a very small difference between an insole and no insole, as shown by the unrescaled deviations (Table 4) and by the size of the bars which correspond to the rescaled deviations (Figure 2). Without defining any specific limit for a ‘positive/negative deviation’ (this approach ensures that all individual differences are accounted for but does not give any size of the effect), the extension insole induced a more extended running pattern for nine participants (50%), while the flexion insole induced a more flexed running pattern for eight participants (44%). Notably, only two participants (11%) reported both a more extended running pattern with the extension insole and a more flexed running pattern with the flexion insole (∆ overall; Figure 2). The highly variable individual responses to the extension and flexion insoles underline the intricate nature of insole interventions and emphasize the importance of personalizing insole treatments. These individual variations further highlight the necessity for future research to investigate how personal differences affect the effectiveness of extension and flexion insoles in running biomechanics. Such variability echoes common findings in the literature and contributes to the ongoing debate surrounding the efficacy of insoles in addressing various pathologies. For instance, two systematic review and meta-analysis reported conflicting evidence in terms of treatment effectiveness (Rasenberg et al., 2018; Whittaker et al., 2018). The first study concluded that there is moderate-quality evidence that insoles are effective at reducing pain in the medium term, however it is uncertain whether this is a clinically important change (Whittaker et al., 2018). The second study concluded that insoles are not superior for improving pain and function compared with sham or other conservative treatment (Rasenberg et al., 2018). Multiple factors might contribute to this variability. First, the diversity of components used in different studies could play a role because an agglomeration of properties, not just one specific property, can influence the behavior of materials (Rome, 1991). Second, inter-practitioner variability is a major factor in custom-made insole intervention because the insole effects were shown to be practitioner-dependant (Chevalier and Chockalingam, 2012). Third, the inability to detect significant group effects could be due to variation in individual subject behaviour. As an example, Stacoff et al. (2000) reported that differences in eversion and tibial rotations between subjects were significantly larger (up to 10°) than between several medial foot orthotic conditions (1°–4°). This can be explained by the paradigms postulated by Nigg et al. (Nigg et al., 2015; Nigg et al., 2017) stating that the use of footwears (insoles herein) should allow healthy runners to maintain their preferred movement path. Hence, caution should be taken with alterations in biomechanical variables representing the running pattern, since they could be in both directions (positive and negative). Altogether, these factors suggest that the prescription of insoles is a difficult task and that methods must further be developed to test and assess these effects (Nigg et al., 2003).

Several limitations were identified in this study and should be considered when interpreting the findings. Firstly, participants wore their own running shoes during testing, which could be confounding our results. Given that differences in footwear characteristics can underpin differences in running biomechanics (Sinclair et al., 2016), using a standardized shoe might have led to different study outcomes in terms of running biomechanics. Nonetheless, recreational runners are more comfortable wearing their own shoes (Hébert-Losier et al., 2020), and show individual responses to novel footwear (Tam et al., 2016; Hébert-Losier et al., 2020) and cushioning properties (Tung et al., 2014). Additionally, previous research has shown that attaching reflective markers on the shoe surface or directly on the skin can influence biomechanical outcomes (Arnold and Bishop, 2013). However, creating holes in the shoes to attach these markers can lead to structural deformation of athletic footwear, potentially affecting in-shoe foot kinematics (Arnold and Bishop, 2013). This is particularly relevant to our research, where participants wore their own shoes, potentially introducing variability in how the holes and resultant deformations impacted the outcomes. Moreover, only acute effects of insoles were examined in the present study. It remains unclear if longer usage of these insoles would have altered the running biomechanics and therefore influenced the observed results. However, extending the run duration (e.g., running for 5 min and capturing several 30-s intervals) could have yielded a more comprehensive dataset, enhancing within-subject comparisons. Future studies could investigate how longer usage may impact running biomechanics. Furthermore, the design of the insoles, while guided by two key principles, lacks a standardized approach. The creative freedom given to the podiatrist during the design process introduces potential variability, as this approach can differ among practitioners. This highlights the non-systematic nature of insole design, reinforcing that the effects of insoles may be practitioner-dependent, as shown in previous studies (Chevalier and Chockalingam, 2012). Nonetheless, this study represents an initial step in exploring the effects of customized extension- and flexion-based insoles. Future research should aim to compare insoles designed by different podiatrists to establish a more systematic approach. Besides, the present study focused, by purpose, on a relatively simple analysis based on two global (DF and ∆COM) and one local ( $t_{\max.\;\text{pron}}$ ) variables to introduce the concept of extension- and flexion-based insoles. While this approach allowed for an initial exploration, more comprehensive analyses involving joint kinematics, forces, and moments are necessary to gain deeper insights. These additional variables could have provided a more complete understanding of the effects of insole interventions on running biomechanics. Additionally, the absence of pressure mapping data limited the ability to assess how the insoles influenced foot pressure distribution and mechanics. This technology could have provided valuable insights into the effects of insoles locally (on foot function and foot biomechanics). Future research should address these gaps and explore the more complex interactions between local and global running variables, incorporating joint data and pressure mapping to provide a deeper understanding of insole interventions. Finally, the sample size of 18 participants, with only four females, was relatively homogeneous, which limits the generalizability of the findings. Although this study adhered to a similar sample size as other research in the field (Wilkinson et al., 2018; Van Alsenoy et al., 2021), a larger and more heterogeneous sample would have allowed for more robust conclusions. A more diverse sample, particularly one with a more balanced representation of sex, could have provided a better understanding of any sex-specific differences in the effects of the insoles on running biomechanics. Future investigations should explore the influence of sex as it is well-established that females exhibit structural differences, potentially resulting in variations in running mechanics (Ferber et al., 2003) and a distinct impact of foot insoles.

## Conclusion

In conclusion, the present study did not find conclusive evidence supporting the anticipated effects of the extension and flexion insoles on running biomechanics. The novel insoles, designed to promote either extension or flexion during running, did not exhibit the expected impact on running mechanics. The highly variable individual responses to these insoles highlight the complexity of insole interventions. This study underscores the challenges associated with such interventions, emphasizing the need for a deeper understanding of individual variability, practitioner-dependent effects, and the biomechanical mechanisms involved in insole design.

## Data Availability

The raw data supporting the conclusions of this article will be made available by the authors, without undue reservation.
